# The Goal of Carbon Peaking, Carbon Emissions, and the Economic Effects of China’s Energy Planning Policy: Analysis Using a CGE Model

**DOI:** 10.3390/ijerph20010165

**Published:** 2022-12-22

**Authors:** Haisheng Hu, Wanhao Dong

**Affiliations:** 1 Business School, University of Shanghai for Science and Technology, Shanghai 200093, China; 2 School of Public Finance & Administration, Shanghai Lixin University of Accounting and Finance, Shanghai 201209, China

**Keywords:** energy policy, nonfossil energy, carbon emissions, economic effect, carbon peaking, CGE

## Abstract

This study focuses on the effects of China’s carbon peaking policy, investigating how to balance nonfossil energy consumption and coal consumption to achieve China’s carbon peaking policy goal. The research applies the recursive dynamic computable general equilibrium model to simulate the impact of China’s energy planning policies using five scenarios to analyze the carbon emissions and economic effects of China’s energy planning policy from the perspectives of energy use, carbon emissions, the macroeconomy, and institutional income. The simulation results indicate that to achieve the goal of carbon peaking by 2030, the annual installed capacity of nonfossil energy must reach 112.29 gigawatts, and average annual coal consumption in the China 15th Five-Year Plan and 16th Five-Year Plan should be reduced by 20 million and 40 million tons, respectively, which will result in the proportion of nonfossil energy in primary energy consumption reaching about 25%. Limiting coal consumption will slow economic growth, whereas increasing the installed capacity of nonfossil energy will stimulate economic growth. The combined policies will have a significant impact on reducing carbon emissions and achieving the carbon peaking goal and will also offset the adverse effects of such policies on the macroeconomy.

## 1. Introduction

China announced the goal of reaching peak carbon dioxide emissions by 2030. This includes goals of a significant decline in energy consumption per unit of GDP; CO_2_ emissions per unit of GDP dropping by more than 65% compared with the 2005 level; the proportion of nonfossil energy consumption reaching around 25%, with the total installed capacity of wind and solar power reaching over 1200 gigawatts according to *Working Guidance for Carbon Dioxide Peaking and Carbon Neutrality in Full and Faithful Implementation of the New Development Philosophy*. Scholars have conducted research regarding when China will reach carbon peak [1,2]. Most studies use mathematical methods, such as the Kaya Inequality method [3] and the Monte Carlo simulation method [1,4] to simulate the realization of carbon peak; however, as a significant, continuously evolving, impact factor, energy policy requires further attention.

The Chinese government’s policy emphasizes that the key approach for the nation to achieve carbon dioxide peak is to implement new energy sources, such as wind and solar power. When the total installed capacity of wind power and solar power reaches over 1200 gigawatts, can the goal of “nonfossil energy accounting for about 25% of primary energy consumption” be achieved? Furthermore, can the goal of reaching peak carbon dioxide emissions in 2030 be achieved? How many gigawatts of wind power and solar power should be installed if China cannot achieve the 2030 goal? Scholars’ research on carbon emissions reduction focuses on the development and implementation of renewable energy [5,6], the development of nuclear power [7], the implementation of ethanol and biodiesel [8], and the coal capacity cut policy [9,10].

If China is unable to achieve the carbon peaking goal by 2030 by increasing the installed capacity of nonfossil energy, how can this goal be achieved? One possible answer is reducing coal consumption. According to *China Statistical Yearbook 2021*, coal consumption accounted for 56.8% of China’s primary energy consumption in 2020. The carbon emissions coefficient of coal is extremely high. Reducing the amount and proportion of coal consumption in the primary energy supply, and applying a combined policy of increasing nonfossil energy, and reducing coal consumption to achieve carbon peaking in 2030 may be a sound policy choice. If such combined policies are adopted, how will other energy consumption change? Will it seriously affect China’s economic growth? This article will attempt to answer these important questions.

There are three approaches used in this research. First, nonfossil energy is embedded into the general equilibrium theoretical analysis framework. Most existing research on carbon emissions examines the impact of fossil energy, such as coal, oil, and natural gas, on carbon emissions. Considering the substitution between energy sources, this study attempts to measure the substitution effect of nonfossil energy on fossil energy to analyze its indirect impact on carbon emissions. Second, several simulation scenarios are analyzed and compared with policy goals to provide a basis for policy formulation. Third, the dynamic impact of energy consumption on major economic variables is measured. The majority of previous studies assert that GDP scale is an important factor affecting energy consumption structure. Using the recursive dynamic computable general equilibrium (CGE) model, this study attempts to analyze the impact of energy consumption structure on socioeconomic variables while analyzing the impact of energy consumption on carbon emissions.

The remainder of this study is arranged into four sections. Section 2 presents a literature review of previous research on energy consumption and carbon emissions. Section 3 constructs the DCGE analysis model of energy policy, using base data from China’s input–output table. Section 4 presents the energy policy simulation scenarios investigated. Section 5 analyzes the simulation results of various policies, compares the advantages and disadvantages of the policies, and finally draws conclusions.

## 2. Literature Review

Focusing on China’s carbon peaking policy and its goals, this study simulates and analyzes potential changes in China’s total energy consumption and consumption structure from 2021 to 2035, including their impact on carbon emissions and the macroeconomy. The research includes four considerations of energy consumption structure analysis, the influencing factors of carbon emissions, the impact of energy policy on the macroeconomy, and the validity of the energy consumption prediction model.

### 2.1. Analysis and Optimization of Energy Consumption Structure

Scholars have undertaken numerous studies analyzing energy consumption structure. There are four key elements that influence the structure of energy consumption. The first is the effect on GDP. In [11], the authors constructed an extended logarithmic mean Divisia index model, identifying economic development as one of the main factors of energy consumption structure change. The second is the effect of industrial structure. In [12], the authors asserted that China’s energy structure and energy consumption could be optimized by lowering the rate of secondary industry and increasing the rate of tertiary industry. The above research is extremely valuable and insightful for analyzing the impact of economic growth on energy consumption structure. Subsequently, changes in the energy consumption structure also have an impact on macroeconomic outcomes, such as economic growth, a research field that must be expanded.

### 2.2. The Impact Factors of Carbon Emissions

Carbon emissions can be influenced by numerous attributes, and energy structure is one of these considerations. Xu et al. [13] and Tang et al. [14] found energy consumption structures to have a negative impact on carbon emissions at each stage of China’s development. In [15,16], the authors analyzed how to achieve the 2030 CO_2_ emission reduction targets for China’s industrial sector and China’s non-fossil energy development with its 2030 CO_2_ reduction targets. In [17], the authors analyzed the driving factors of carbon emissions from China’s mining sector. Xu et al. [18] asserted that the low-carbon energy consumption structure adjustment had an increasingly important impact on carbon emissions, and economic structure is a non-negligible factor. Zhang et al. [19] demonstrated that economic structure became increasingly carbon intensive, which increased carbon emissions. As for renewable energy substitution, Chiu and Chang [20] used a panel threshold regression model to examine the relationship between renewable energy supply and carbon emissions, finding that only when the proportion of renewable energy supply reaches 3.3889% of all energy supplied will the dilemma between carbon emissions and economic growth be solved. Dong et al. [21] determined that short- and long-run bidirectional causality exists between renewables and carbon emissions, and renewables can mitigate carbon emissions. Other factors influence carbon emissions, including GDP per capita [22,23,24,25], industrialization level [5], urbanization level [26], and technological progress [27]. The insightful observations of these scholars established energy consumption as the most important factor affecting carbon emissions. The researchers assume that renewable energy can directly replace traditional energy, and the development of renewable energy is the key path to reducing carbon emissions; however, quantitative measurement of the substitution effect of renewable energy on traditional energy must be deeply studied.

### 2.3. The Impact of Energy Policy on the Macroeconomy

Previous research regarding energy consumption and economic growth can be summarized into four hypotheses of growth, conservation, feedback, and neutrality [28]. The growth hypothesis asserts that energy access has an important influence in promoting economic development [29]. For example, evidence has been revealed in favor of energy-led growth by Iyke [30] for Nigeria, Odhiambo [31] for Brazil and Uruguay, Shahbaz et al. [32] for China, Ouedraogo [33] for 15 ECOWAS countries, and Ghosh [34] for India. The conservation hypothesis suggests that economic growth can promote energy consumption. The unidirectional causality running from economic growth to energy consumption has been supported by multiple empirical studies, such as Odhiambo [31] for Ghana and Cote d’Ivoire. The interdependent relationship between energy consumption and economic growth is the premise of the feedback hypothesis, and the neutrality hypothesis asserts that there is no causality between energy consumption and economic development. The two-way causal feedback relationship between energy consumption and economic growth has been deeply studied; however, findings remain contradictory. Nevertheless, most of these studies confirm the existence of this factor and offer valuable insights. The quantitative measurement of the impact degree of the two-way causal relationship is the field of future research.

### 2.4. Forecasting Model

The ability to predict a country’s future energy consumption structure objectively and accurately is of considerable importance and can provide a significant reference for policymakers to formulate energy development strategies and related economic policies. Several scholars have used different energy prediction models to study related issues, which can be divided into three types: statistical models, machine learning methods, and gray models. Peng et al. [35] combined gray and risk assessment models to forecast the energy security in China, concluding that the nation’s coal consumption will decrease dramatically from 2020 to 2029. Zhang et al. [36] established a novel flexible gray multivariable model to forecast energy consumption according to data from three main provinces in China. Shi et al. [37] applied a combined algorithm using a self-adaptive differential evolution algorithm and a support vector optimization algorithm to predict coal consumption, demonstrating that China’s coal consumption will be between 2.195 billion tons and 3.699 billion tons by 2030. Wei et al. [38] used comprehensive time series forecasting models, revealing a sharp drop in coal consumption and a huge potential for the advancement of nonfossil energy by 2030 in China. He and Lin [39] constructed an ADL-MIDAS model to estimate energy demand. Zhao and Luo [40] employed a vector error correction model to forecast fossil energy consumption structure. Ding et al. [41] predicted nuclear energy consumption in China and the United States using an optimized structure adaptive gray model. He et al. [42] proposed two Box–Cox transformation quantile regression methods to determine future energy consumption trends in Anhui province in China. Yuan et al. [43] used an autoregressive integrated moving average and a gray model (GM (1,1)) to predict primary energy consumption in China. Cui et al. [44] dynamically revealed energy consumption in Shanxi province using vector autoregression (VAR) and STIRPAT models. Although the above research used various models, few studies have employed a CGE model to predict energy consumption structure. Bohlmann et al. [45] employed a regional CGE model to examine the long-term regional economic effects of changing the electricity-generation mix toward less coal within South Africa. Nong et al. [46] applied the GTAP-E-Power model to examine the impact of Vietnam’s ratification of the Paris agreement. Qi et al. [47] analyze the economic impacts of differentiated carbon reduction targets by using a two-region CGE model in Tianjin Municipal City of China.

Summarizing the above literature, the research on the influencing factors of energy consumption structure and carbon emissions are very insightful. Many scholars used gray prediction and statistical empirical models to analyze the impact of energy consumption on carbon emissions. All of this research provides the background for this study. 

The first innovation of this paper is the measurement of the renewable energy substitution effect and research on the impact of energy consumption and energy structure on the macroeconomy which is a new research field. The study will provide energy policy suggestions for China to achieve the 2030 carbon peaking goal, and also serve as a reference for the formulation of relevant energy policies in other countries.

The second innovation of this paper is innovation in the production module. The energy capital factors are subdivided as follows: oil, coal, natural gas, and nonfossil energy. Nonfossil energy factor is divided into four capital factors, those are nuclear power, hydropower, wind power, and solar power. The subdivision of factors provides a basis for subsequent energy policy simulation, and it is also an innovation for the CGE model. 

The third innovation is that carbon emission is added as the result of simulation in order to observe the impact of different energy policies on carbon emission in the CGE model. Which makes this general equilibrium method appropriate to study the impact of energy policy, especially the carbon peaking policy.

## 3. Theoretical Model

This study adopts a DCGE model, which is a macroeconomic model based on the assumptions of factor flow and market clearing, particularly including energy factors, it is suitable to analyze the impact of energy policy on carbon emissions. The DCGE have advantages compared to other models for this research question. The model has three specific characteristics to address the research problems. 

(1) In the production module, capital factors are divided into energy capital factors and other capital factors. In the statistical specifications of primary energy in the China Statistical Yearbook, the factors of energy capital are subdivided into four sub-factors of coal, oil, natural gas, primary power, and other energy. Similarly, with reference to the statistical specifications of power generation installed capacity in the China Statistical Yearbook, there are primary energy power and other energy power, and primary energy power includes hydropower, nuclear power, wind power, and solar power. These improvements are designed to suit the analysis of China’s carbon peaking policy. (2) According to the industry statistical specifications of energy consumption in the China Energy Statistical Yearbook, the model includes 15 production activity sectors, the primary industry is agriculture, the secondary industry includes 11 industries, and the tertiary industry includes three industries. This subdivision method not only maintains the unity of data, but also provides an excellent data basis for the simulation of China’s carbon peaking policy. (3) In the output module, in addition to the output of various commodities, CO_2_ emissions are included to measure the impact of different energy policies on environmental protection.

Referring to the research results of Chang [48] and Sancho [49], the core equation of the DCGE model is presented below. This DCGE model included 74 equations. Due to space limitations, only the primary equations are listed. All variables in the equation have time attributes. According to common expressions of the DCGE method, the subscript t of all variables is omitted.

### 3.1. Production Module

The significant difference between the model used in this study and other dynamic CGE models is the integration of an added value function and energy factors in the production module. In this study, the value-added function is in the form of the Cobb–Douglas function. The value-added is composed of various factor inputs, as shown in Equation (1). The equilibrium prices of various factors are shown in Equations (2)–(4). The subdivision of each factor is as previously noted.

(1) $$QVA_{a}=ad_{a}*QLD_{a}^{alphalab_{a}}*QKOD_{a}^{alphacapo_{a}}*\prod_{n}QKED_{i,a}^{alphacape_{i,a}}$$

(2) $$WL*QLD_{a}=alphalab_{a}*PVA_{a}*QVA_{a}$$

(3) $$WKO*QKOD_{a}=alphacapo_{a}*PVA_{a}*QVA_{a}$$

(4) $$WKE_{i}*QKED_{i,a}=alphacape_{i,a}*PVA_{a}*QVA_{a}$$

where QVA is the added value of capital, labor and energy factors; a is sectors’ activity; QLD is labor demand; PVA is the price of added value; QKOD is the demand of capital; i represents various types of primary energy, including coal, oil, natural gas, primary power, and other energy, including hydropower, nuclear power, wind power, and solar power; $QKED_{i,a}$ is the demand for various types of primary energy elements in sector a; WL is the price of labor factors; WKO is the price of capital factors; $WKE_{i}$ is the price of the energy i; ad is the production technology level of added value, alphalab is the elasticity coefficient of labor; alphacapo is the elasticity coefficient of capital; and alphacape is the elasticity coefficient of energy.

### 3.2. Income Distribution Module

Enterprise income comes from the capital and energy factor compensation owned by the enterprise. Government transfer payments are also enterprise income. The enterprise income functions are shown as Equations (5) and (6):

(5) $$YENT=shif_{entk}\cdot WKO\cdot QKOSAGG+shif_{ente}\cdot WKE\cdot QKESAGG+transfr_{entg}$$

(6) $$ENTSAV=\left(1-ti_{ent}\right)YENT$$

where YENT is enterprise income; QKOSAGG is the aggregate supply of other capital factors; QKESAGG is the aggregate supply of energy factors; $shif_{entk}$ is the share of capital income allocated to the enterprise; $shif_{ente}$ is the share of energy income allocated to the enterprise; $transfr_{entg}$ represents the income from government transfer payments to the enterprise; ENTSAV is enterprise savings; and $ti_{ent}$ is the enterprise income tax rate.

The change in energy supply quantity will affect income distribution, also changing the prices of capital and labor factors; therefore, the compensation of household labor and capital factors will also change, affecting household income. The household income functions are shown as Equations (7) and (8):

(7) $$YH=WL\cdot QLSAGG+WKO\cdot shif_{hko}\cdot QKOSAGG+WKE\cdot shif_{hke}\cdot QKESAGG+cdist\cdot CY+tranfr_{hg}$$

(8) $$YD=YH-wt\cdot WL\cdot QLSAGG-rt\left(WKO\cdot shif_{hko}\cdot QKOSAGG+WKE\cdot shif_{hke}\cdot QKESAGG+cdist\cdot CY\right)$$

where YH and YD are household income and disposable income, respectively; $shif_{hko}$ is the share of capital income distributed to households; $shif_{hke}$ is the share of energy income distributed to households; CY is corporate profits; cdist is the share of corporate profits distributed to households; $tranfr_{hg}$ is the transfer payment from the government to households; and wt and rt are the residents’ labor income tax and capital income tax rates, respectively.

YG is government revenue, which comes from all kinds of taxes. The change in energy supply will have an impact on the general equilibrium of the economy; thus, the change in indirect government tax revenue. Due to the fluctuation of household and enterprise income, these two types of income are the direct tax base, which will also lead to fluctuation of government income. The government revenue function is shown as Equation (9):

(9) $$\begin{array}{c} YG=\sum_{a}tbus_{a}\cdot PA_{a}\cdot QA_{a}+wt\cdot WL\cdot QLSAGG+rt(WKO\cdot shif_{hko}\cdot QKOSAGG+\\ WKE\cdot shif_{hke}\cdot QKESAGG+cdist\cdot CY)+ti_{ent}*YENT \end{array}$$

where $tbus_{a}$ represents other production tax rates, PA represents the price of aggregate output, QA represents the quantity of aggregate output of domestic production activities.

### 3.3. Macro Closure

Based on different economic theories, the closure of the DCGE macro model may adopt neoclassical closure, Keynesian closure, and Lewis closure. Referencing Chang [48], this study constructs dynamic module closure on the basis of neoclassical closure.

The commodity market has reached a general equilibrium. Under the condition of a given price and exchange rate, the quantity of goods is endogenous. There are no restrictions on the import and export of goods, and the commodity market is cleared. The commodity market equilibrium is shown in Equation (10):

(10) $$QQ_{c}=\sum_{a}QINT_{ca}+QH_{c}+QINV_{c}+QG_{c}$$

where $QQ_{c}$ is the supply of commodity c in the domestic market; QINT is the intermediate input in the production of commodity c; $QH_{c}$ is households’ demand for goods; $QINV_{c}$ is the investment demand for commodity c; and $QG_{c}$ is the government’s fiscal expenditure demand for commodity c.

The factor market has reached a general equilibrium. With the clearing of the labor market, the aggregate demand for labor is equal to the aggregate supply of labor, which is shown in Equation (11):

(11) $$\sum_{a}QLD_{a}=QLSAGG$$

With the clearing of the capital factor market, the aggregate demand for capital is equal to the aggregate supply:

(12) $$\sum_{a}QKOD_{a}=QKOSAGG$$

With the clearing of the energy factor market, the aggregate demand for capital is equal to the aggregate supply:

(13) $$\sum_{a}QKED_{i,a}=QKESAGG_{i}$$

Capital investment has reached a general equilibrium. $QKOSAGG_{t}$ is the t period capital supply, which is equal to the capital factor supply of the previous period ($QKOSAGG_{t-1}$), plus the capital investment of the previous period ($INV_{t-1}$) minus depreciation ($DEP_{t-1}$).

(14) $$QKOSAGG_{t}=QKOSAGG_{t-1}+INV_{t-1}-DEP_{t-1}$$

From the above theoretical mechanism analysis, the demand elasticity of various sectors for various types of energy varies, and changes in different types of energy supply will directly impact CO_2_ emissions, which will also lead to changes in added value and GDP. Changes in energy consumption will be transmitted to the production module. The production efficiency of enterprises will change, impacting commodity prices and demand, leading to changes in macroeconomic variables, such as economic growth, enterprises income, household income, and government revenue.

## 4. Base Data and Parameter Calibration

### 4.1. Base Data

The base data of the DCGE model is a Social Accounting Matrix (SAM). This study prepared the SAM using China’s energy consumption characteristics as the base data for the DCGE model to analyze the carbon peaking policy. The SAM data are from the 2017 China input–output table, the China Statistical Yearbook 2021, the China Tax Yearbook 2020, the China Energy Statistical Yearbook 2020, and the China Environmental Statistical Yearbook 2020, issued by the National Bureau of Statistics.

SAM preparation requires the decomposition and aggregation of various sector data to address research problems. Referencing Hu et al. [50] and considering the current circumstances of China’s energy production and consumption and the availability of data, the SAM used in this study includes 15 sectors, the primary industry is agriculture, the secondary industry is divided into 11 industries, and tertiary industry is divided into 3 industries. This research using cross entropy method adjust the SAM in order balance the column and row [51]. The macro SAM shown as Table 1.

### 4.2. Parameter and Parameter Calibration

In this study, the important parameters are usage hours of nonfossil energy power generation equipment and CO_2_ emissions. This is also one of the significant aspects of this study.

Data for the parameters of usage hours of nonfossil energy power generation equipment are according to the National Power Industry Statistics (2020) issued by the National Energy Administration and the China Energy Big Data Report (2021) issued by China Power Media Corporation, this study calculated equipment use hours presented in Table 2. The annual power generation of nonfossil energy is calculated based on the installed capacity and equipment use hours.

Data for the parameters of CO_2_ emissions for various energy sources references to Zhou and Hong [52], as well as data from the 2006 Intergovernmental Panel on Climate Change Guidelines for National Greenhouse Gas Inventories and the China Energy Statistical Yearbook 2020. The CO_2_ emissions coefficients of various energy sources in this study are presented in Table 3.

(15) $$E_{CO_{2}}=\sum_{i}E_{i}\cdot S_{i}\cdot Ef_{i}$$

where $E_{CO_{2}}$ is the aggregate amount of CO_2_ released through energy consumption, $E_{i}$ is energy consumption, $S_{i}$ is the conversion coefficient of various energy sources into standard coal, and $Ef_{i}$ is the CO_2_ emissions coefficient.

The parameters, such as the substitution elasticity in CES production function, Armington elasticity, and CET elasticity, reference the research of Chang [48]. The other production function parameters in this CGE model, such as share parameters and scale parameters, are calculated and calibrated using the base subdivided SAM table data.

## 5. Scenario Settings

Considering that China’s energy policy predominantly focuses on nonfossil and coal energy, this study establishes scenarios from these two perspectives. Scenarios 1 and 2 concern nonfossil energy development policy. Scenarios 3 and 4 concern coal development planning policy. Scenario 5 combines scenarios 2 and 4.

All the scenario settings are aimed at the policy goals of the Chinese government. Those goals from the *Working Guidance for Carbon Dioxide Peaking and Carbon Neutrality in Full and Faithful Implementation of the New Development Philosophy* issued by China National Development and Reform Commission, the *Action Plan for Carbon Dioxide Peaking Before 2030* issued by China State Council, and *China Nuclear Energy Development Report 2022* by the China Nuclear Energy Industry Association.

### 5.1. Benchmark Scenario

The benchmark scenario setting is based on 2020 data. There is no energy policy to impact the general equilibrium, and energy consumption is determined by the market. We then simulate the impact of energy policy on carbon emissions and economic development from 2021 to 2035 to examine the shock to general equilibrium.

### 5.2. Scenario 1

According to the classification in the China Statistical Yearbook (2021), nonfossil energy primarily includes hydropower, nuclear power, wind power, and solar power generation. The nonfossil energy policy in this study is also described from these four aspects:

Hydropower planning. According to *Action Plan for Carbon Dioxide Peaking Before 2030,* approximately 40 gigawatts of additional hydropower capacity will be installed during the 14th and 15th Five-Year Plan periods, while a renewable energy system largely based on hydropower will be established in Southwestern China. This study assumes that during the period from 2021 to 2030, the annual installed capacity of hydropower is 8 gigawatts to achieve the goal of the 15th Five-Year Plan. Considering the severe circumstances of carbon emissions and the coherence of energy policy, it is assumed that China will continue to increase the installed capacity of hydropower by 8 gigawatts every year from 2031 to 2035.

Nuclear power planning. According to the website of China’s National Atomic Energy Agency, the installed capacity of nuclear power in operation and under construction will reach 200 gigawatts by 2035. In 2020, China’s nuclear power installed capacity was 49.89 gigawatts. Based on this estimation, it is assumed that the annual installed capacity will reach 10.01 gigawatts in 2021 to about 200 gigawatts by 2035.

Wind power planning and solar power generation planning. According to the Chinese government’s goal, the total installed capacity of wind and solar power will reach more than 1200 gigawatts by 2030. In 2020, the installed capacities of wind and solar power generation in China were 281.53 and 253.43 gigawatts, respectively. Accordingly, it is assumed that the installed capacity of wind and solar power generation will increase by 35 and 31.51 gigawatts, respectively, from 2021 to 2030, and the total installed capacity of wind and solar power generation will reach 1200 gigawatts by 2030. Considering that new energy power generation, such as wind and solar power generation, is the focus of China’s subsequent development, it is assumed that wind and solar power generation will maintain an annual installed capacity of 35 and 31.51 gigawatts, respectively, from 2031 to 2035.

The above parameters regarding the development of nonfossil energy is set as scenario 1; that is, the annual installed capacity for hydropower is 8 gigawatts, the annual installed capacity for nuclear power is 10.01 gigawatts, the annual installed capacity for wind power is 35 gigawatts, and the annual installed capacity for solar power is 31.51 gigawatts.

### 5.3. Scenario 2

Considering current policy circumstances in China, we then adjusted scenario 1. Scenario 2 considers the effects of different policies.

In recent years, China has been increasing investment in wind and solar power generation projects. By 2030, the actual total installed capacity of wind and solar power generation is expected to be higher than the minimum goal of 1200 gigawatts. For wind energy, according to the Beijing Declaration on wind energy issued at the 2020 Beijing International Wind Energy Conference (CWP 2020), China’s annual installed capacity of wind energy will increase by more than 50 gigawatts by 2030. Based on this, this study assumes that the average annual new installed capacity of wind energy in China from 2021 to 2035 is 50 gigawatts. The long-term plan for the development of China’s solar installed capacity has not been released. The average annual newly installed capacity from 2016 to 2020 is 44.28 gigawatts, with a rapid development speed. This study assumes that the annual newly installed capacity after 2021 will continue to maintain the development of the past five years, with an annual newly installed capacity of 44.28 gigawatts.

Accordingly, scenario 2 is set as follows. The growth of hydropower and nuclear power remains the same as scenario 1. Assuming that the average annual growth of wind and solar power from 2021 to 2035 is 50 gigawatts and 44.28 gigawatts, respectively, the total installed capacity of wind and solar power needs to reach about 1478 gigawatts by 2030.

### 5.4. Scenario 3

According to China’s Action Plan for Carbon Dioxide Peaking Before 2030, one of the key tasks is *promoting coal substitution as well as transformation and upgrading,* wherein the government will accelerate the pace in reducing coal consumption, strictly and rationally limiting the increase in coal consumption over the 14th Five-Year Plan period, and further phasing it out in the 15th Five-Year Plan period.

In 2020, the increment of coal consumption was 15.83 million tons of standard coal. Based on this, this study set scenario 3. During the 14th Five-Year Plan period (2021–2025), annual new coal consumption will be reduced by 2.64 million tons of standard coal compared with the previous year. During the 15th Five-Year Plan period (2025–2030), annual coal consumption will decrease by 10 million tons of standard coal compared with the previous year. During the 16th Five-Year Plan period (2031–2035), annual coal consumption will decrease by 20 million tons of standard coal compared with the previous year.

### 5.5. Scenario 4

Considering the gradual reduction in coal consumption during the 15th Five-Year Plan period, which directly affects China’s total energy consumption and carbon emissions scale, to examine the effect of the reduction in carbon consumption of different scales, scenario 4 is set. During the 14th Five-Year Plan period, annual new coal consumption will be reduced by 2.64 million tons of standard coal compared with the previous year (the same as scenario 3). During the 15th Five-Year Plan period, annual coal consumption will decrease by 20 million tons of standard coal compared with the previous year. During the 16th Five-Year Plan period, annual coal consumption will decrease by 40 million tons of standard coal compared with the previous year.

### 5.6. Scenario 5

Considering that China’s energy development plan includes both nonfossil energy and coal development plans, this study sets a comprehensive simulation scheme in scenario 5, which simulates scenarios 2 and 4 simultaneously.

The specific settings of the five scenarios are presented in Table 4.

## 6. Simulation Results Analysis

This study simulates the above five scenarios for energy planning policies, the observation variables are energy usage, carbon emissions, and the macroeconomy.

### 6.1. Simulation Results of Energy Usage

**Figure 1:**
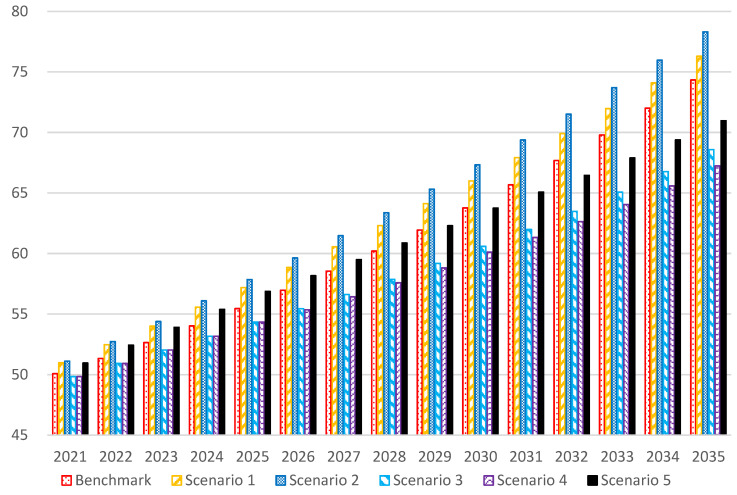
Simulation of energy consumption in different scenarios (100 million tons of standard coal).

Figure 1 presents the benchmark scenario in which China’s total energy consumption will reach 5.546 billion tons of standard coal by 2025, 6.376 billion tons of standard coal by 2030, and 7.434 billion tons of standard coal by 2035.

The simulation results of scenario 1 demonstrate that with the increase in installed capacity of nonfossil energy, total energy consumption will increase by 173 million tons, 223 million tons, and 196 million tons in 2025, 2030, and 2035, respectively.

With further expansion of the installed capacity of wind and solar power generation (scenario 2), total energy consumption also increases, with an increase of 66 million tons, 133 million tons, and 201 million tons in 2025, 2030, and 2035, respectively, compared with scenario 1.

The simulation results of scenario 3 indicate that after limiting the consumption of coal, total energy consumption will significantly decline, and will be more obvious over time. Total energy consumption will be reduced by 112 million tons, 317 million tons, and 575 million tons in 2025, 2030, and 2035, respectively, compared with the benchmark scenario.

With the further reduction in coal consumption in 2025–2030 (scenario 4), the energy consumption in 2030 and 2035 will be further reduced by 48 and 135 million tons, respectively, compared with scenario 3, which can effectively reduce both coal consumption and total energy consumption.

From the simulation results of scenario 5, the total energy consumption in 2025, 2030, and 2035 will reach 5.687, 6.376, and 7.097 billion tons, respectively, an increase of 141 million tons, the same level, and a decrease of 337 million tons compared with the benchmark scenario.

Based on the above, the conclusion is that rapidly increasing the installed capacity of nonfossil energy will not reduce total energy consumption in the short term, whereas reducing coal consumption can quickly decrease total energy consumption.

At the UN climate ambition summit on December 12, 2020, the Chinese government announced the goal of the proportion of nonfossil energy in primary energy consumption reaching 25% by 2030. In the simulation results of six scenarios, the proportion of nonfossil energy in primary energy consumption in 2030 is 18.79%, 21.70%, 23.35%, 19.95%, 20.14%, and 24.65%, respectively.

Regarding the goal of 25% of nonfossil energy by 2030, the simulation results demonstrate two primary findings.

➀ When the total installed capacity of wind power and solar power reaches 1200 gigawatts, the goal of the proportion of nonfossil energy of 25% cannot be achieved. The total installed capacity of wind and solar power reaches 1478 gigawatts. The installed capacity of hydropower and nuclear power should reach 450 gigawatts and 150 gigawatts, respectively, by 2030 to achieve the goal of a 25% proportion of nonfossil energy.
➁ China should make further efforts to restrict coal consumption. From 2025 to 2030, the consumption of standard coal should be reduced by 10 million tons per year from the previous year. The simulation results of scenario 5 also indicate that the proportion of coal, oil, and natural gas in energy consumption will be 43.42%, 19.54%, and 12.39% by 2030, respectively. The proportion of coal, oil, natural gas, and nonfossil energy consumption will be further optimized, at 36.19%, 19.82%, 16.35%, and 27.64% by 2035, respectively.

**Figure 2:**
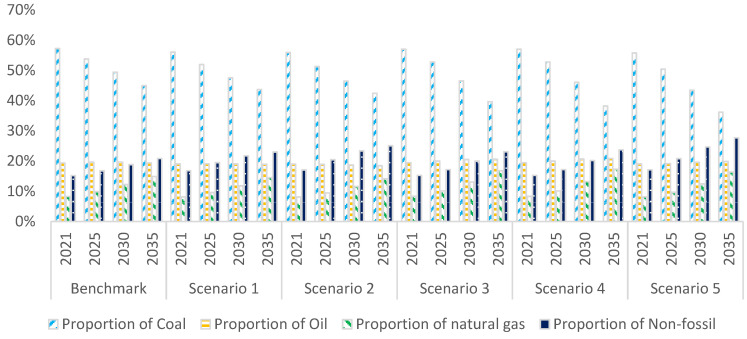
Simulation of energy consumption composition in different scenarios.

Figure 2 shows that only under the combined scenario of nonfossil energy planning and restricted coal consumption (scenario 5), can the goal of nonfossil energy announced by the Chinese government be achieved.

### 6.2. Simulation Results of Carbon Emissions

**Figure 3:**
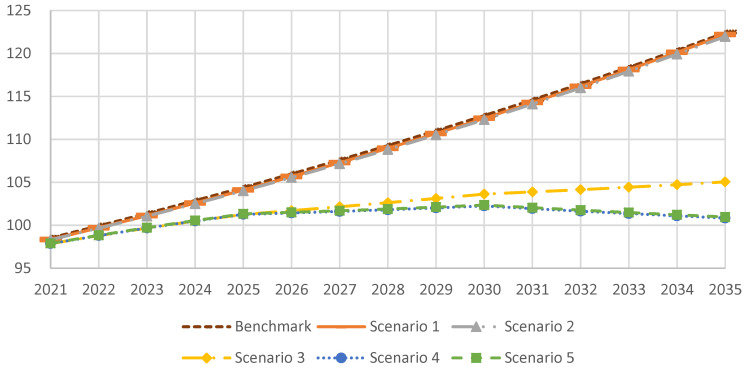
Carbon emissions simulation results of China’s energy consumption under different scenarios (100 million tons).

The simulation results are presented in Figure 3. The carbon emissions data curves of the benchmark scenario and scenarios 1 and 2 almost coincide. In these three scenarios, the carbon emissions of China’s energy consumption will be 11.278 billion tons, 11.246 billion tons, and 11.229 billion tons by 2030, respectively, and subsequent carbon emissions will continue to increase, indicating that an increase in nonfossil energy investment will not affect the trend of carbon emissions if coal consumption is not limited.

In scenario 3, limiting coal consumption will significantly reduce carbon emissions. The carbon emissions of energy consumption will be 10.363 billion tons by 2030 and 10.505 billion tons in 2035, indicating that reducing carbon emissions by 20 million tons per year during the 16th Five-Year Plan period will not achieve the goal of carbon peaking.

The carbon emissions curves of scenarios 4 and 5 also almost coincide, reaching a peak value of 10.225 billion tons and 10.236 billion tons by 2030, respectively, and carbon emissions will decline to 10.084 billion tons and 10.097 billion tons by 2035, respectively. The results show that when the annual decline of coal consumption reaches 40 million tons, the goal of a carbon peak can be successfully achieved.

The substitution effect of the increase in nonfossil energy input on fossil energy is relatively limited. The realization of carbon peaking by 2030 requires the limitation of coal consumption, necessitating the implementation of coal energy planning policies.

**Figure 4:**
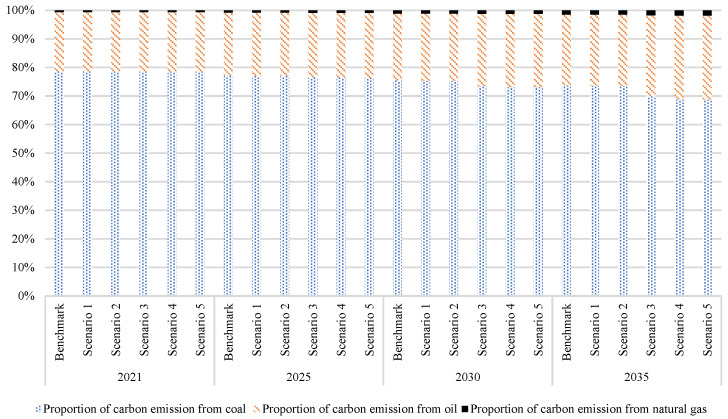
Carbon emissions composition of China’s energy consumption under different scenarios.

Coal is the primary source of carbon emissions in China. Rapid substitution of nonfossil energy for coal energy while reducing coal consumption is the key path for China’s achievement of carbon peaking. Most of the carbon emissions from China’s energy consumption are caused by coal consumption, which can be seen in Figure 4. In the benchmark scenario, coal consumption accounted for 57.23% of total energy in 2021, but the proportion of carbon emissions was 78.56%. The reason was primarily due to the lower standard coal conversion coefficient of coal compared with other energy sources, resulting in a higher carbon emission coefficient. In scenario 5, coal consumption accounts for 43.42% of total energy, and carbon emissions account for 73.05% in 2030.

The increase in nonfossil energy can provide new sources of energy when reducing coal consumption. This makes it possible to achieve the goal of carbon peaking for China.

Natural gas has high energy calorific value and a relatively low carbon emissions coefficient. In scenario 5, natural gas accounts for 12.39% of energy consumption in 2030, but the proportion of carbon emissions is only 1.30%; therefore, increasing the development and supply of natural gas can also effectively reduce carbon emissions to achieve the carbon peaking goal.

### 6.3. Simulation Results of Macroeconomic Variables

**Figure 5:**
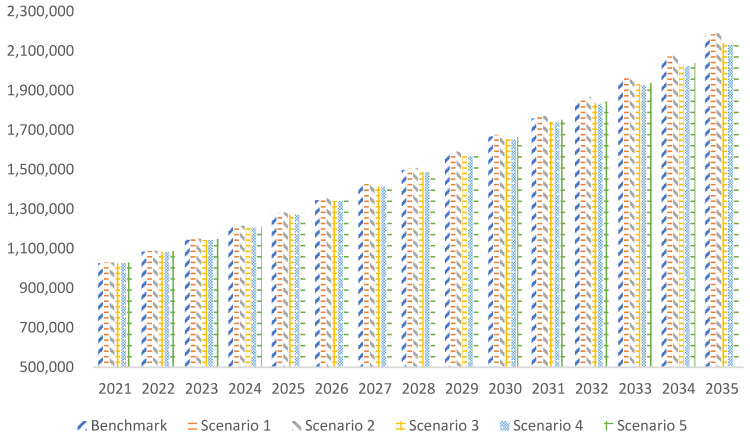
China’s GDP growth under different scenarios (100 million RMB).

Increasing the installed capacity of nonfossil energy will stimulate GDP growth, whereas limiting coal consumption will slow GDP growth. This trend can be seen in Figure 5.

In the benchmark scenario, China’s GDP will reach 166.64 trillion RMB in 2030. When the installed capacity of nonfossil energy is increased, the GDP in 2030 in scenarios 1 and 2 will increase by 0.47% and 0.70%, respectively, compared with the benchmark scenario.

Increasing the investment in nonfossil energy projects will significantly promote the GDP. When limiting coal consumption, the GDP in 2030 in scenarios 3 and 4 decreased by 0.79% and 0.92%, respectively, compared with the benchmark scenario, indicating that energy consumption limitations will slow China’s GDP growth.

The GDP in 2030 is about 166.32 trillion RMB in scenario 5, with a decrease of 0.19% compared with the benchmark scenario. The investment in the installed capacity of nonfossil energy will offset the negative impact of coal consumption restrictions. China’s carbon dioxide emissions per 10,000 RMB of GDP was 3.00 tons in 2005, whereas China’s carbon dioxide emissions per 10,000 RMB of GDP in 2030 was 0.62 tons in scenario 5, with a decrease of 79.49% compared to 2005. This will achieve the goal announced by the Chinese government that the share of nonfossil energy consumption will reach around 25%, and carbon dioxide emissions per unit of GDP will drop by more than 65% by 2030 compared with the 2005 level, successfully achieving carbon dioxide peaking.

**Figure 6:**
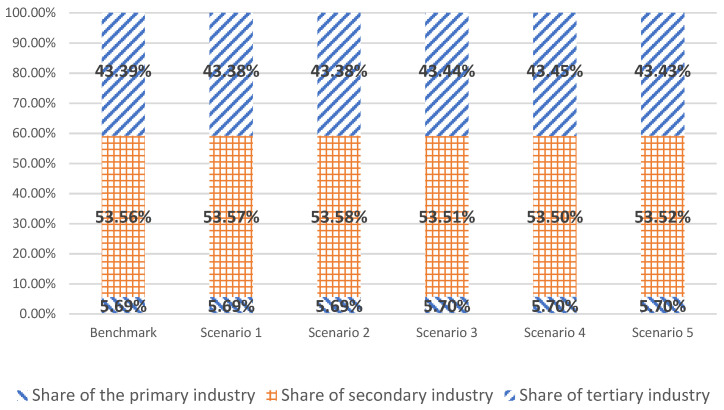
Share of China’s industries in the GDP in 2030 under different scenarios.

The above energy planning policies have a heterogeneous impact on the industry, which can be seen in Figure 6. The impact of the energy planning policies on the primary industry is relatively slight. Under various scenarios, the share of the primary industry will remain at about 5.70% in 2030. Increasing the installed capacity of nonfossil energy will increase the share of the secondary industry in the GDP, which is the same trend in the simulation results of scenarios 1 and 2. Cutting coal consumption and strictly and rationally limiting the increase in coal consumption will reduce the share of secondary industry in the GDP. The simulation results of scenarios 3 and 4 show this trend. Increasing the installed capacity of nonfossil energy has a weak impact on the tertiary industry, and the share of the tertiary industry in the GDP in scenarios 1 and 2 has decreased slightly.

Cutting coal consumption and strictly and rationally limiting the increase in coal consumption also has a weak impact on the tertiary industry. In scenarios 3 and 4, the share of the tertiary industry in the GDP increased due to the relatively large decline of the secondary industry. In scenario 5, the share of the tertiary industry in 2030 increases slightly compared with the benchmark scenario.

**Figure 7:**
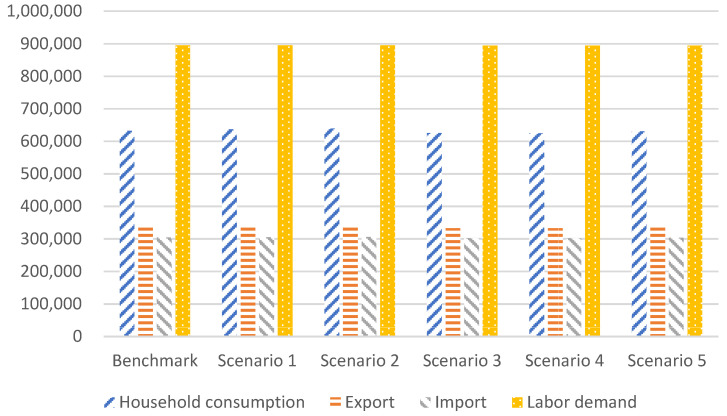
Change in major macro variables in China in 2030 under different scenarios (100 million RMB).

The impact of the above energy planning policies on other major macroeconomic variables is almost the same as that on GDP in different simulation scenarios. As shown in Figure 7.

Household consumption is a main observation variable in this study. Under the benchmark scenario, household consumption will be about 63.26 trillion RMB in 2030. Scenarios 1 and 2 are policies to increase the installed capacity of nonfossil energy. Household consumption will increase by 0.66% and 0.99%, respectively, compared with the benchmark scenario. Scenarios 3 and 4 are policies to limit the increase in coal consumption. Household consumption decreases by 1.13% and 1.32%, respectively, compared with the benchmark scenario.

Scenario 5 is a compromise that comprehensively considers the combination of increasing nonfossil energy and reducing coal consumption. In this scenario, household consumption will decrease by 0.29% by 2030 compared with the benchmark scenario, presenting an acceptable scheme. The combined policy can reduce the adverse impact on the macroeconomy while reducing carbon emissions.

This study also observes variables such as import and export and labor demand, which also provide an observation perspective for tradeoff policies. Under different scenarios, the impact of policies on variables such as import and export and labor demand elicits a similar trend to that of household consumption.

### 6.4. Simulation Results of Institutional Income

**Figure 8:**
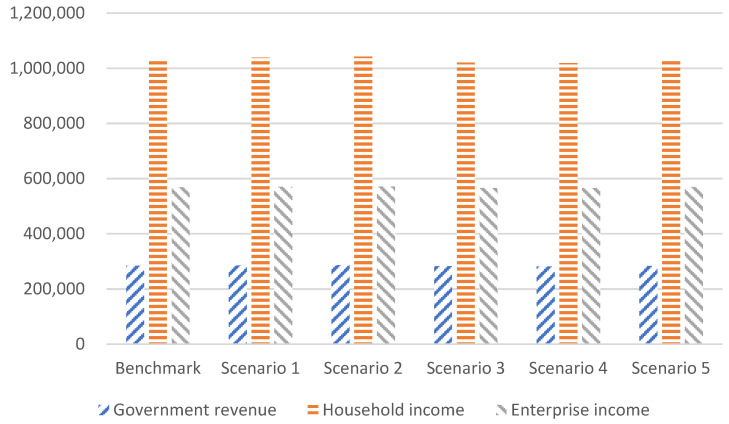
Institutional income in different scenarios in 2030 (100 million RMB).

Government revenue includes multiple sources of tax revenue. Household income includes capital income distributed to households, energy income distributed to households, corporate profits distributed to households, and transfer payments from the government to households. Enterprise income comes from capital factor compensation, energy factor compensation owned by the enterprise, and government transfer payments. The institutional income under different scenarios is shown in Figure 8.

China’s government revenue in the benchmark scenario will be 28.35 trillion RMB in 2030; the government revenue in scenarios 1 and 2 will increase by 0.42% and 0.62%, respectively, in 2030, compared with the benchmark scenario. The simulation results show that increasing the installed capacity of nonfossil energy will stimulate the growth of government revenue. China’s government revenue in scenarios 3 and 4 decreased by 0.59% and 0.68%, respectively, in 2030, compared with the benchmark scenario; the simulation results demonstrate that limiting coal consumption will reduce government revenue. Government revenue in scenario 5 will decrease by 0.04% in 2030, compared with the benchmark scenario, it shows that the combined energy planning policy will reduce carbon emissions with a weak negative impact on government revenue.

China’s household income in the benchmark scenario will be 103.21 trillion RMB in 2030; the household income in scenarios 1 and 2 will increase by 0.65% and 0.97%, respectively, compared with the benchmark scenario. Increasing the installed capacity of nonfossil energy will stimulate the growth of household income. Household income in scenarios 3 and 4 decreased by 1.13% and 1.31%, respectively, in 2030, compared with the benchmark scenario. Limiting coal consumption will reduce household income. The household income in scenario 5 decreased by 0.29% in 2030, compared with the benchmark scenario. This demonstrates that combined energy planning policies also reduced household income.

Enterprise income will be 56.83 trillion RMB in 2030 in the benchmark scenario. Enterprise income in scenarios 1 and 2 increased by 0.38% and 0.57%, respectively, compared with the benchmark scenario. Increasing the installed capacity of nonfossil energy will expand enterprise investment and increase enterprise income. Enterprise income in scenarios 3 and 4 decreased by 0.41% and 0.48%, respectively, in 2030, compared with the benchmark scenario. Limiting coal consumption will increase enterprise production costs and reduce enterprise income. Enterprise income in scenario 5 increased by 0.09% in 2030, compared with the benchmark scenario. This suggests that combined energy planning policies will not reduce enterprise income and will also stimulate the slight growth of enterprise income.

## 7. Conclusions and Policy Implications

This study constructed a DCGE model, using China’s input–output table as base data for this approach, and simulating five energy planning policies of increasing the installed capacity of nonfossil energy and limiting coal consumption. The simulation results are energy utilization, carbon emissions effect, and economic effect under the shock of the policy. Several conclusions can be drawn from the above analysis.

Increasing the installed capacity of nonfossil energy and strictly and rationally limit increases in coal consumption are the key to achieving the carbon peaking goal of the share of nonfossil energy in primary energy consumption reaching about 25% by 2030 announced by the Chinese government. This goal cannot be achieved by increasing the installed capacity of nonfossil energy or limiting coal consumption alone. According to the simulation results of the above combined energy policy in this study, the total installed capacity of wind and solar power generation must reach 1478 gigawatts; China’s total energy consumption will reach 6.376 billion tons of standard coal, and nonfossil energy accounts for about 24.65% of primary energy consumption by 2030, almost achieving the goal of the share of nonfossil energy.

Most previous research assumes that renewable energy directly replaces traditional energy. This paper measures the substitution effect of renewable energy on traditional energy. Coal is the primary source of carbon emissions in China’s energy consumption. The increase in nonfossil energy has a weak substitution effect for fossil energy from the perspective of carbon emissions; therefore, increasing the installed capacity of nonfossil energy combined with strict control of coal consumption is the key for China to achieve the carbon peaking goal by 2030. According to the simulation results of scenario 5, carbon peaking will be achieved in 2030, with emissions from China’s energy consumption of 10.236 billion tons. The combination of this policy includes an annual installed capacity of hydropower of 8 gigawatts, an annual installed capacity of nuclear power of 10.01 gigawatts, an annual installed capacity of wind power of 50 gigawatts, and an annual installed capacity of solar power of 44.28 gigawatts. Annual new coal consumption decreased by 2.64 million tons of standard coal compared with the previous year from 2021 to 2025, annual coal consumption decreased by 20 million tons of standard coal compared with the previous year from 2026 to 2030, and annual coal consumption decreased by 40 million tons of standard coal compared with the previous year from 2031 to 2035.

Increasing the installed capacity of nonfossil energy will boost energy investment and stimulate GDP growth, whereas limiting coal consumption will restrain enterprises’ energy demand and slow GDP growth; therefore, the combined energy planning policy of increasing nonfossil energy while restraining coal consumption can effectively hedge the adverse impact of such policies on the economy.

Different from existing research that focused on the impact of economic growth on energy consumption structure, this paper simulated the impact of energy consumption structure on economic growth. China’s GDP will reach 166.32 trillion RMB in 2030 in scenario 5, which is only 0.19% lower than the benchmark scenario. China’s carbon dioxide emissions per 10,000 RMB of GDP is 0.62 tons in 2030, which is 79.49% lower than that in 2005. This will meet the goal announced by the Chinese government that “by 2030, China’s carbon dioxide emissions per unit of GDP will be more than 65% lower than that in 2005”.

The trend of the impact of energy planning policies on other major macroeconomic variables is almost the same. Increasing the installed capacity of nonfossil energy will stimulate increased household consumption, import and export, and labor demand. Limiting coal consumption will slow residents’ consumption, import and export, and labor demand. The combined energy planning policy can hedge this impact on the macroeconomy, while reducing carbon emissions.

Increasing the installed capacity of nonfossil energy will increase government revenue, household income, and enterprise income, and the increase in household income is the most significant, followed by government revenue and enterprise income. Limiting coal consumption will reduce government revenue, household income, and enterprise income. There are different impacts on institutions’ income when combined energy planning policies are implemented. Government revenue and household income in scenario 5 will decrease by 2030 when the combined energy planning policy is implemented, and it will stimulate enterprise income.

In conclusion, China’s combined energy policy of increasing the installed capacity of nonfossil energy and limiting coal consumption will achieve the three policy goals of peak carbon, the share of nonfossil energy in primary energy consumption reaching 25%, and CO_2_ emissions per unit of GDP will be more than 65% lower than that in 2005. In this research, various policies are combined for trial, which has its advantages. The results show that the combination of policies has a positive effect on reducing carbon emissions and can effectively neutralize adverse impacts on economic growth and reduce the negative effects of policy macroeconomic variables and institutional income.

## Figures and Tables

**Table 1:** China macro SAM 2017 (billion RMB).

	Expenditures											
Receipts	(I)	(II)	(III)	(IV)	(V)	(VI)	(VII)	(VIII)	(IX)	(X)	(XI)	(XII)
Activities (I)		226,039.8										225,880.1
Commodities (II)	143,451.3				32,042.7		12,375.1		36,181.3	531.0	16,385.2	240,806.9
Labor (III)	42,133.3											42,133.3
Capital (IV)	30,957.5											30,797.8
Households (V)			42,133.3	3302.7			6807.8					52,243.8
Enterprises (VI)				27,654.8								27,654.8
Government (VII)	1354.0							13,770.1				13,905.5
Tax (VIII)	9362.3				1196.1	3211.7						13,905.5
Savings–Investment (IX)					19,005.0	24,443.1	−5277.4				−1458.4	36,552.6
Stock Change (X)									531.0			531.0
Rest of World (ROW) (XI)		14,926.8										14,926.8
Total (XII)	226,039.8	240,806.9	42,133.3	30,957.5	52,243.8	27,654.8	13,905.5	13,770.1	36,712.3	531.0	14,926.8	

Note: The first column and first row are the same. Due to space limitations, in the first row, only numbers are shown.

**Table 2:** Annual usage hours of nonfossil energy power generation equipment in China from year 2017 to 2020 (hours).

Energy Type	2017	2018	2019	2020
Hydro energy	3463	3494	3697	3827
Nuclear energy	6925	6591	7394	7453
Wind energy	1807	1986	2083	2073
Solar energy	747	1018	1169	1281

**Table 3:** The carbon emissions coefficient of various energy sources.

Energy Type	Conversion Standard Coal Coefficient	CO_2_ Emissions Coefficient
Coal (kg/kg)	0.7057	1.90
Oil (kg/kg)	1.4264	3.02
Natural gas (kg/m^3^)	1.3140	2.16
Nonfossil power (kg/kwh)	29.3827	0

**Table 4:** Scenario settings for China’s energy policy simulations.

Scenario	Policy	Year 2021–2025	Year 2026–2030	Year 2031–2035
**Scenario** 1	Nonfossil energy development policy	⯎ Hydropower, the annual installed capacity of hydropower is 8 gigawatts. ⯎ Nuclear power, the annual installed capacity is 10.01 gigawatts. ⯎ Wind power, the annual installed capacity is 35 gigawatts. ⯎ Solar power, the annual installed capacity is 31.51 gigawatts.		
**Scenario** 2		⯎ Hydropower, the annual installed capacity of hydropower is 8 gigawatts (the same as scenario 1). ⯎ Nuclear power, the annual installed capacity is 10.01 gigawatts (the same as scenario 1). ⯎ Wind power: the annual installed capacity is 50 gigawatts. ⯎ Solar power: the annual installed capacity is 44.28 gigawatts.		
**Scenario** 3	Coal energy development policy	The annual new coal consumption decreased by 2.64 million tons of standard coal compared with the previous year.	The annual coal consumption decreased by 10 million tons of standard coal compared with the previous year.	The annual coal consumption decreased by 20 million tons of standard coal compared with the previous year.
**Scenario** 4		The annual new coal consumption decreased by 2.64 million tons of standard coal compared with the previous year (the same as scenario 3).	The annual coal consumption decreased by 20 million tons of standard coal compared with the previous year.	The annual coal consumption decreased by 40 million tons of standard coal compared with the previous year.
**Scenario** 5	Combined policy	Scenario 2 combine with Scenario 4		

## Data Availability

The data presented in this study are available on request from the corresponding author.
